# Self-employment, educational attainment, and hypertension among Black women and men

**DOI:** 10.3389/fepid.2022.991628

**Published:** 2022-09-26

**Authors:** Caryn N. Bell, Carlos D. Tavares, Jessica L. Owens-Young, Roland J. Thorpe

**Affiliations:** ^1^Department of Social, Behavioral, and Population Science, Tulane University School of Public Health and Tropical Medicine, New Orleans, LA, United States; ^2^Department of Anthropology and Sociology, Lafayette College, Easton, PA, United States; ^3^Department of Health Studies, American University, Washington, DC, United States; ^4^Department of Health, Behavior and Society, Bloomberg School of Public Health, Johns Hopkins University, Baltimore, MD, United States

**Keywords:** self-employment, educational attainment, hypertension, Black Americans, gender

## Abstract

**Background:**

Self-employment is generally associated with better health outcomes and educational attainment can shape self-employment. Yet, Black Americans are less likely to be self-employed and analyses of self-employment and health among Black Americans are few. The aim of this study was to determine how educational attainment moderates the associations between self-employment and hypertension among Black adults.

**Methods:**

Using data from the 2007–2018 National Health and Nutrition Examination Survey, participants who self-identified as non-Hispanic Black (*n* = 2,855) were categorized as (1) employees with no self-employment income, (2) employees with self-employment income, or (3) having full-time self-employment. Modified Poisson regressions and multiplicative interaction terms were used to determine whether educational attainment moderated the associations between self-employment and measured hypertension (i.e., 140/90 mm Hg or anti-hypertensive medication).

**Results:**

Most participants were employees with no self-employment income (81.9%), but 11.8% were employees reporting some self-employment income and 6.3% were self-employed full-time. About two in five (40.9%) had hypertension. Having full-time self-employment was associated with lower risk of hypertension compared to those who were employees (risk ratio = 0.82, 95% confidence interval = 0.67–0.98), and educational attainment moderated the associations among Black men such that part-time self-employment was associated with high rates of hypertension among Black men who had not completed high school.

**Conclusions:**

These results suggest that full-time self-employment is associated with lower risk of hypertension among Black adults, but that being an employee with some self-employment income may elevate rates of hypertension among Black men depending on educational attainment. Future studies should assess pathways between self-employment and hypertension by educational attainment among Black women and men.

## Introduction

More than half of Black Americans have blood pressure ≥130/80 mm Hg or report taking anti-hypertensive medication and only one in four Black Americans who take medication have controlled blood pressure (1–3). Studies have shown that individual-level discrimination is associated with hypertension among Black Americans (4–10). However, structural measures of racism such as racial segregation and racial differences in social and residential contexts have also been connected to hypertension and racial inequities (11–19). Structural racism may also shape how work-related factors impact hypertension (20). For example, John Henryism is a concept studied since the 1980s and is described as a disposition toward work characterized by “high-effort and commitment to hard work.” Scholars describe how John Henryism among Black Americans is a result of experiences and perceptions of racial inequities in economic outcomes and opportunities due to structural racism in the U.S. (21) and John Henryism has been linked to several stress-related health outcomes including hypertension (21–24).

Self-employment is another work-related determinant of hypertension and is understudied among Black Americans. The pursuit of self-employment may be viewed as a means of resistance to both workplace racism as well as broader systemic racism that limits economic opportunities for Black Americans (25, 26). Studies on self-employment and hypertension have had mixed results (27–30). Self-employment can be associated with increased stress and negative impacts on various health outcomes (31). Yet, some research suggests that self-employment is associated with better health outcomes because of higher overall job control and autonomy (28, 29, 32, 33). Though Black Americans are less likely to be self-employed than White Americans overall (25, 34, 35), a study by Narain and Skrine Jeffers (29) finds that odds of hypertension are lower among self-employed Black Americans compared to those who are employees. The study found that the negative association between self-employment and hypertension only applied to high-income women whereas the negative association was observed only among men with lower incomes (28). This suggests that there are gendered and socioeconomic factors that can impact the association between self-employment and hypertension.

Socioeconomic inequities have been assessed to explain racial differences in self-employment rates (36–38). However, educational attainment does not predict self-employment among Black Americans (39). Educational attainment does influence the characteristics of self-employment with potential implications for its impact on hypertension. College-educated adults are more likely to be entrepreneurs (e.g., be involved in a business start-up or nascent entrepreneurship) than non-high school graduates (40). A 2021 study by Solomon et al. examined the intersections of self-employment, educational attainment, and work characteristics, finding that job satisfaction was not associated with educational attainment among those who were self-employed. However, the study found that self-employed adults with higher educational attainment had less autonomy, less variety in their work, and greater qualitative demands (41). These studies suggest that self-employment characteristics and experiences related to stress, job control, and job satisfaction may vary by educational attainment. Because self-employment affects factors like stress, job autonomy and control, and job satisfaction (27–30, 42). this likely has implications for health outcomes such as hypertension. Educational attainment could impact the pathways that lead to the negative association between self-employment and hypertension observed in other studies of Black Americans.

Additionally, a growing number of Americans report having a side hustle (43). New forms of “gig” work (typically less structured work arrangements facilitated through online platforms) have contributed to this increase (44). Adults pursue part-time self-employment to increase income (45) and it can result in increases in empowerment (46). Highly educated adults are more like to pursue self-employment in the form of start-ups (40). Because of historic wealth inequities between Black and White adults (47–54) that have been linked to lower rates of Black entrepreneurship (38, 55), these pursuits may begin as part-time self-employment. Thus, the association between hypertension and self-employment among Black Americans should be interrogated by educational attainment accounting for the possibility of part-time as well as full-time self-employment.

The aim of this study is to determine whether the association between employment categories and hypertension is moderated by educational attainment among Black adults. It is hypothesized that a negative association between self-employment and hypertension will be observed among Black adults with less education and a positive association between self-employment and hypertension will be observed among more educated Black adults. These analyses will contribute to our understanding of the nuanced ways in which work characteristics impact hypertension among Black Americans.

## Methods

### Data

The National Health and Nutrition Examination Survey (NHANES) is a nationally representative dataset of the health, nutrition, and health behaviors on civilian, non-institutionalized adults and children (56). The NHANES uses an in-home interview to collect data on health history, behaviors, and other risk factors. A medical examination is also conducted on a subsample. Using a stratified, multistage probability sampling design, the NHANES oversamples low-income individuals, those who self-identify as Hispanic, non-Hispanic Black or African American, and non-Hispanic Asian, as well as adults over age 80 years old. Data is collected over a 2-year period and a new nationally representative sample is collected every 2 years. The NHANES began collecting detailed data on income and self-employment in 2007. To obtain a large number of non-Hispanic Black respondents, this study combined data from 2007 to 2018. A sample of 2,855 women and men who identified as non-Hispanic Black and who did not have missing data for any variables were included in analyses.

### Variables

The dependent variable was hypertension. A dichotomous variable was created to indicate whether the respondent had hypertension or not. Respondents have their blood pressure measured up to four times during the medical examination. Dropping the first blood pressure measurement, average systolic and diastolic blood pressure were calculated (57). Respondents were also asked whether they were currently taking prescribed anti-hypertensive medication. Respondents with measured blood pressure ≥140/90 mm Hg or who were currently taking anti-hypertensive medication were given a value of “1.” A value of “0” was given to respondents with blood pressure <140/90 mm Hg who did not report currently taking anti-hypertensive medications.

The main independent variable was employment category. Respondents who were currently working were asked to describe their job or work situation and responses included: (1) an employee of a private company, business, or individual for wages, salary, or commission, (2) a federal government employee, (3) a state government employee, (4) a local government employee, (5) self-employed in own business, professional practice or farm, or (6) working without pay in family business or farm. Those who were employed by a company, business, individual, the federal government, a state government, or a local government were categorized as employees. Those who responded that they were self-employed in their own business, professional practice, or farm were categorized as “full-time self-employed.” Additionally, all respondents were also asked if they received any income from self-employment. Because a proportion of respondents who were employees of a company, business, individual, the federal government, a state government, or a local government also reported receiving self-employment, a separate category was assessed. Therefore, a three-category variable was created with the following categories: (1) employee with no self-employment income, (2) employee with self-employment income, and (3) full-time self-employed.

The moderating variable was educational attainment. Respondents reported the highest level of education that they completed. Educational attainment was categorized as follows: not a high school graduate, high school graduate or recipient of a GED, some college or Associate's degree, and ≥Bachelor's degree.

Covariates were included in statistical analyses because they may confound the associations between the independent and dependent variables (27, 28, 30, 33, 58–61). *Age* was measured continuously. *Marital status* was categorized as currently married or living with partner, formerly married (e.g., separated, divorced, or widowed), and never married. *Household income* was measured by the income-to-poverty ratio. Reported annual income relative to the poverty line based on household size was calculated and included as a continuous variable. *Body mass index (BMI)* was calculated as weight (kg) divided by height (m^2^) and dichotomized at BMI ≥ 30 kg/m^2^. *Alcohol consumption* was measured by asking respondents if they have consumed alcohol in the last year. A dichotomous variable was created to represent current drinkers. *Smoking status* was assessed by asking respondents if they ever smoked at least 100 cigarettes and if they currently smoke cigarettes. A dichotomous variable was created to represent current smokers. *Physical inactivity* was assessed by asking respondents how often they participated in moderate or vigorous physical activity. A dichotomous variable was created such that those who responded that they never participated in moderate or vigorous activity were categorized as physically inactive.

### Statistical analyses

Descriptive statistics were calculated for all analytical variables by gender (e.g., male or female). Modified Poisson regression models were used because the prevalence of hypertension was not rare (about 40%) (62). First, the associations of employment categories, educational attainment, and gender with hypertension were assessed in Model 1. Using multiplicative interaction terms, the potential moderation of the associations between employment categories with hypertension by educational attainment and gender were assessed in Model 2 because gender differences in the associations between self-employment and health observed in a previous study (28). A significant three-way interaction suggests that the potential moderation of the association between employment category and hypertension by educational attainment varied by gender. As such, remaining analyses were assessed within gender categories also using multiplicative interaction terms. The associations between employment category, educational attainment, and hypertension (Model 1) were assessed among women and among men. Then, the potential moderation of the association between employment category and hypertension by educational attainment (Model 2) were assessed among women and among men. Following the procedure recommended by the National Center for Health Statistics, all analyses used Taylor-linearization procedures for the complex multistage sampling design (56). A weight variable was created to account for the combining of multiple years of NHANES (63–65) and the use of data from the medical examination subsample. A *p*-value of ≤ 0.50 was used to indicate significance and associations from regression models include 95% confidence intervals. The 0.05 significance level is demonstrated when the 95% confidence interval does not include 1.00. All statistical analyses were conducted in STATA Version 17 (StataCorp LP, College Station, TX).

## Results

All variables included in analyses for this study are displayed by gender in Table 1. There were gender differences in age, marital status, income, working hours, self-rated health, obesity, physical inactivity, smoking, and drinking. There were also gender differences in employment categories (*p* < 0.001). About 8% of Black men were full-time self-employed, while about 5% of Black women had full-time self-employment. There were gender differences in educational attainment as well (*p* < 0.001). Almost one in five Black women had a Bachelor's degree or more, while about 16% of Black men had completed a 4-year college degree. Forty-two (42.3%) of Black women had hypertension measured as blood pressure ≥ 140/90 mm Hg or currently taking prescribed anti-hypertension medication compared to about 39.3% of Black men (*p* = 0.015).

**Table 1:** Analytical variables by gender among Black adults, National Health and Nutrition Examination Survey (NHANES) 2007–2018.

	**Total**	**Female**	**Male**	
	**N = 2,855**	**N = 1,459**	**N = 1,396**	*p*-value^*^
Age (years), mean ± s.e.	45.0 ± 0.3	45.6 ± 0.4	44.3 ± 0.4	0.003
Marital status, %				
Currently	42.6	36.3	50.5	<0.001
Formerly	23.6	28.9	17.1	
Never	33.8	34.9	32.4	
Household income, %				
<$20,000	24.5	27.1	21.2	<0.001
$20,000–$34,999	21.7	23.0	20.1	
$35,000–$54,999	19.3	18.8	19.9	
$55,000–$74,999	12.1	11.2	13.2	
≥$75,000	22.4	19.9	25.6	
BMI ≥ 30 kg/m^2^, %	47.6	55.3	38.1	<0.001
Current smoker, %	24.8	19.9	31.0	<0.001
Current drinker, %	72.1	63.3	82.7	<0.001
Physically inactive, %	52.9	58.2	46.4	<0.001
Employment category, %				
Employee with no self-employment income	81.9	83.9	79.6	<0.001
Employee with self-employment income	11.8	11.4	12.3	
Full-time self-employment	6.3	4.7	8.1	
Educational attainment, %				
Not a high school graduate	20.0	18.7	21.6	<0.001
High school graduate/GED equivalent	27.1	24.2	30.7	
Some college/Associate's degree	34.7	37.3	31.4	
≥Bachelor's degree	18.2	19.7	16.3	
Hypertension, %	40.9	42.3	39.3	0.015

* p-value demonstrates differences by gender. s.e., standard error; BMI, body mass index.

In Table 2, the associations between employment category, educational attainment, gender, and hypertension are displayed among Black adults. Adjusting for covariates, Black adults who had full-time self-employment had 18% lower relative risk of hypertension compared to employees with no self-employment income in Model 1 [relative risk (RR) = 0.82, 95% CI = 0.68–0.98]. Educational attainment was not associated with hypertension and there were no gender differences. In Model 2, significant three-way interactions between employment category, educational attainment, and gender was observed.

**Table 2:** Associations between employment category educational attainment, gender, and hypertension among Black adults, National Health and Nutrition Examination Survey (NHANES) 2007–2018.

	**Model 1**	**Model 2**
	**RR (95% CI)**	**RR (95% CI)**
Employment category		
Employee with no self-employment income	1.00	1.00
Employee with self-employment income	0.97 (0.85–1.11)	1.53 (0.96–2.43)
Full-time self-employment	0.82 (0.68–0.98)	0.60 (0.35–1.02)
Educational attainment		
Not a HS grad	1.00	1.00
HS grad	0.97 (0.83–1.13)	1.08 (0.86–1.35)
Some college/AA degree	1.02 (0.88–1.18)	1.08 (0.84–1.39)
≥Bachelor's degree	1.01 (0.84–1.22)	1.13 (0.83–1.54)
Female	0.95 (0.85–1.07)	1.09 (0.88–1.35)
Employment category × educational attainment		
Employee with self-employment income × HS grad		0.44 (0.21–0.92)
Employee with self-employment income × some college/AA degree		0.65 (0.35–1.22)
Employee with self-employment income × ≥Bachelor's degree		0.53 (0.26–1.09)
Full-time self-employed × HS grad		1.73 (0.84–3.55)
Full-time self-employed × some college/AA degree		1.06 (0.53–2.11)
Full-time self-employed × ≥Bachelor's degree		1.34 (0.67–2.69)
Employment category × gender		
Employee with self-employment income × female		0.34 (0.14–0.81)
Full-time self-employed × female		1.36 (0.67–2.76)
Educational attainment × gender		
HS grad × female		0.80 (0.62–1.04)
Some college/AA degree × female		0.88 (0.66–1.16)
≥Bachelor's degree × female		0.77 (0.55–1.09)
Employment category × educational attainment × gender		
Employee with self-employment income × HS grad × female		4.14 (1.29–13.25)
Employee with self-employment income × some college/AA degree × female		3.07 (1.11–8.48)
Employee with self-employment income × ≥Bachelor's degree × female		4.29 (1.49–12.40)
Full-time self-employed × HS grad × female		0.67 (0.21–2.14)
Full-time self-employed × some college/AA degree × female		1.01 (0.39–2.62)
Full-time self-employed × ≥Bachelor's degree × female		0.93 (0.37–2.37)

HS, high school graduate/GED equivalent, AA, Associate's. Models adjusted for age, marital status, household income, body mass index ≥ 30 kg/m^2^, physical inactivity, current smoking, and current drinking.

To further assess these interactions, gender-stratified analyses were examined in Tables 3, 4. Among Black women, there was no association between employment category or educational attainment and hypertension (Model 1) as demonstrated in Table 3. There was no interaction between employment category and educational attainment on hypertension among women (Model 2). However, among Black men in Table 4, employment category was associated with hypertension (Model 1). Black men who had full-time self-employment had 23% lower risk of hypertension compared to those who were employees with no self-employment income (RR = 0.77, 95% CI = 0.61–0.96). In Model 2, there was a significant interaction between being an employee with some self-employment income and being a high school graduate among Black men (RR = 0.44, 95% CI = 0.21–0.92).

**Table 3:** Associations between employment category educational attainment, and hypertension among Black females, National Health and Nutrition Examination Survey (NHANES) 2007–2018.

	**Model 1**	**Model 2**
	**RR (95% CI)**	**RR (95% CI)**
Employment category		
Employee without self-employment income	1.00	1.00
Employee with self-employment income	1.01 (0.81–1.27)	0.53 (0.26–1.12)
Full-time self-employed	0.92 (0.68–1.25)	0.84 (0.52–1.36)
Educational attainment		
Not a HS grad	1.00	1.00
HS grad	0.90 (0.74–1.11)	0.87 (0.69–1.10)
Some college/AA degree	1.00 (0.83–1.21)	0.96 (0.77–1.21)
≥Bachelor's degree	0.96 (0.75–1.23)	0.90 (0.69–1.17)
Employment category × educational attainment		
Employee with self-employment income × HS grad		1.77 (0.72–4.34)
Employee with self-employment income × some college/AA degree		1.93 (0.88–4.23)
Employee with self-employment income × ≥Bachelor's degree		2.18 (0.96–4.94)
Full-time self-employed × HS grad		1.14 (0.48–2.71)
Full-time self-employed × some college/AA degree		1.05 (0.51–2.14)
Full-time self-employed × ≥Bachelor's degree		1.20 (0.59–2.43)

HS, high school graduate/GED equivalent, AA, Associate's. Models adjusted for age, marital status, household income, body mass index ≥ 30 kg/m^2^, physical inactivity, current smoking, and current drinking.

**Table 4:** Associations between employment category educational attainment, and hypertension among Black males, National Health and Nutrition Examination Survey (NHANES) 2007–2018.

	**Model 1**	**Model 2**
	**RR (95% CI)**	**RR (95% CI)**
Employment category		
Employee without self-employment income	1.00	1.00
Employee with self-employment income	0.93 (0.77–1.12)	1.52 (0.96–2.40)
Full-time self-employed	0.77 (0.61–0.96)	0.59 (0.34–1.02)
Educational attainment		
Not a HS grad	1.00	1.00
HS grad	1.05 (0.87–1.27)	1.07 (0.85–1.34)
Some college/AA degree	1.05 (0.85–1.29)	1.07 (0.83–1.39)
≥Bachelor's degree	1.10 (0.85–1.42)	1.13 (0.82–1.55)
Employment category × educational attainment		
Employee with self-employment income × HS grad		0.44 (0.21–0.92)
Employee with self-employment income × some college/AA degree		0.66 (0.36–1.23)
Employee with self-employment income × ≥Bachelor's degree		0.53 (0.26–1.09)
Full-time self-employed × HS grad		1.80 (0.88–3.67)
Full-time self-employed × some college/AA degree		1.10 (0.55–2.18)
Full-time self-employed × ≥Bachelor's degree		1.37 (0.48–2.74)

HS, high school graduate/GED equivalent, AA, Associate's. Models adjusted for age, marital status, household income, body mass index ≥ 30 kg/m^2^, physical inactivity, current smoking, and current drinking.

**Figure 1:**
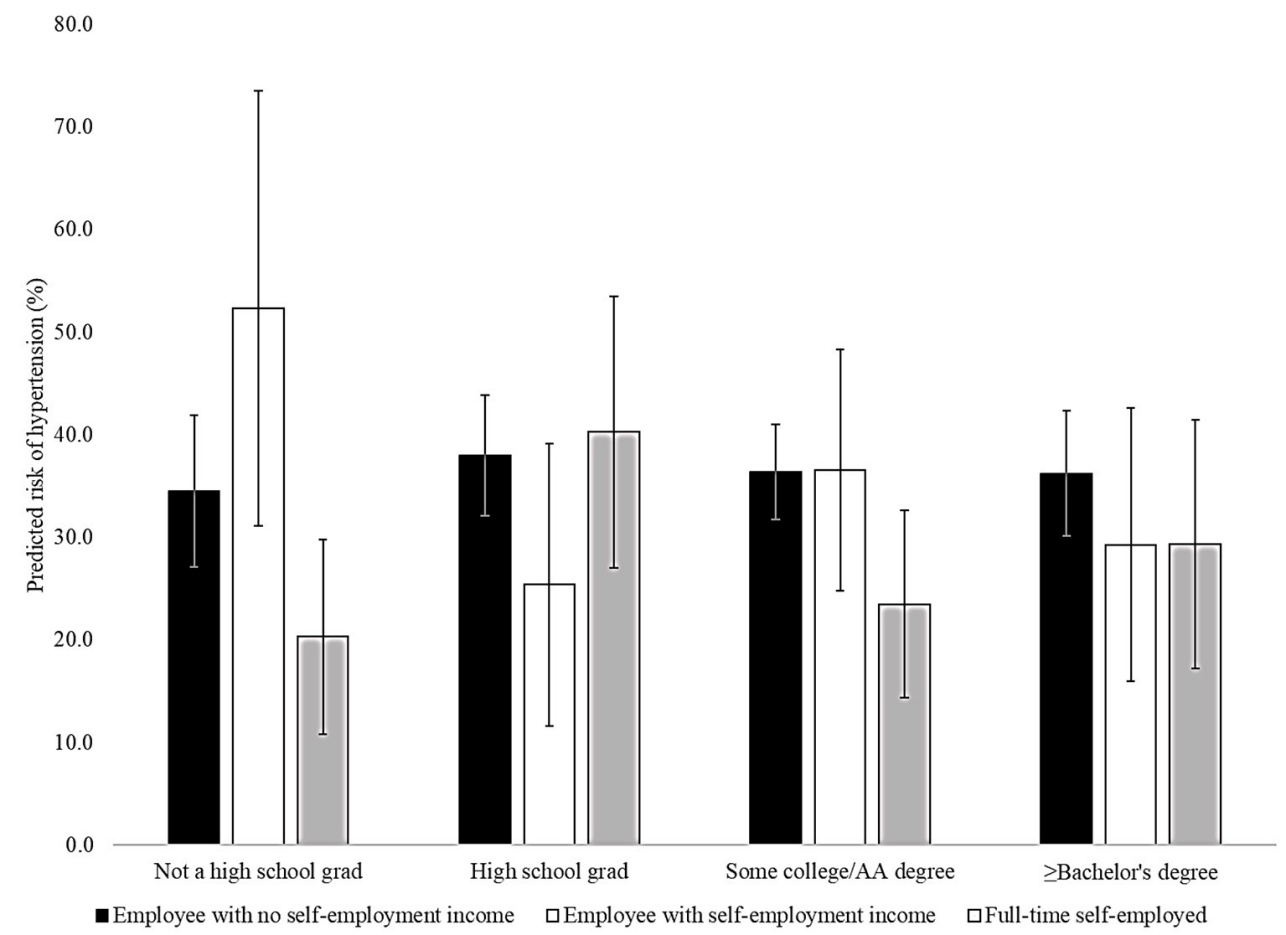
Association between employment category and hypertension by educational attainment in Black males, National Health and Nutrition Examination Survey (NHANES) 2007–2018. AA, Associate's. Models adjusted for age, marital status, household income, body mass index ≥ 30 kg/m^2^, physical inactivity, current smoking, and current drinking.

Figure 1 displays hypertension by employment category and educational attainment among Black men. Among those who had not completed high school, 52.4% of those who were employees but also received self-employment income had hypertension compared to 34.4% among those who were employees with no self-employment income and 20.3% among those who were full-time self-employed. In Black men with more educational attainment, the 95% confidence intervals overlap suggesting that the predicted probabilities of hypertension do not vary by employment category.

## Discussion

Work-related and other socioeconomic factors have been assessed in analyses of hypertension among Black Americans. Full-time self-employment was associated with lower relative risk of hypertension among Black men, but not among Black women. The interaction between educational attainment and employment category on hypertension were significant among Black men and suggest that Black men who have not completed high school and are employees with some self-employment income have higher risk of hypertension. This is particularly important given the recent changes in work and work-related experiences associated with the rise of adults with multiple jobs (43, 66).

The finding that full-time self-employment was associated with lower rates of hypertension agrees with some previous studies (28, 29). However, while Stephan and Roesler (29) and Narain and Skrine Jeffers (28) found lower odds of hypertension and elevated blood pressures among self-employed adults compared to employees, Krittanawong et al. (27) found that those who were self-employed had higher odds of hypertension than those who were employees in a study of self-employment and cardiovascular risk using data from NHANES. There were several key differences compared to the current study that might contribute to this discrepancy. The previous study did not account for household income which may be implicated in the pathways that link self-employment to health outcomes (67). The previous study also included race as a potential confounder and did not conduct race-specific analyses.

It is unclear why risk of hypertension did not vary by employment category among Black women. One previous study assessed the associations between self-employment and self-reported hypertension among Black Americans by gender. The study found that self-employment was associated with lower odds of hypertension among Black men with lower incomes and associated with lower odds of hypertension among Black women with higher income. Therefore, it is likely that household income plays a role in the association between self-employment and hypertension among Black women and not educational attainment.

No previous studies have examined hypertension and adults who are employees with some self-employment income. Though there were no differences in rates of hypertension between employees with self-employment income and employees with no self-employment income overall, educational attainment moderated these associations among Black men. These findings suggest that Black men who are employees with self-employment income but have not completed high school have with higher predicted probability of hypertension. There are several likely explanations for these differences in associations across educational attainment. Self-employment may be characterized by increased stress (31) and this may be particularly acute among Black men with receive self-employment income but are still employees for a company, business, individual, or governmental agency while also having lower educational attainment. For example, lower educational attainment is associated with pursuit of self-employment due to financial needs (68). Thus, the stressful nature of self-employment may be compounded by stress related to financial needs among Black men with lower educational attainment who pursue part-time self-employment (69). Auguste, Roll, and Despard (69) found that, among low and moderate income respondents, access to health insurance plays an important an role in the association between employment type and economic security. Educational attainment is also associated with another self-employment characteristic—entrepreneurship. Adults with lower educational attainment are less likely to pursue self-employment that involves starting a novel business (40). Job control may be a key factor in the health benefits of self-employment (42), and this may not be experienced among Black men with lower educational attainment and who receive self-employment income while still being employees.

Among Black men who did not complete high school, full-time self-employment was associated with lower predicted probability of hypertension than those who received self-employment income while being employed by a company, business, individual, or governmental agency. At higher levels of educational attainment, there were no large differences in predicted probability of hypertension among Black men (see Figure 1). Differences in the experiences of full-time self-employed Black men and those who are employees but receive self-employment income may be salient only among those with less educational attainment.

Educational attainment did not moderate the associations between self-employment and hypertension among Black women. It is possible that other factors are important among Black women. For example, Narain and Skrine Jeffers (28) found that lower odds of hypertension among self-employed Black women were only observed among those with high income. Other studies have examined the role of motherhood, work-life balance, and flexible work schedules among self-employed women (70–72). These factors may be more salient to the relationship between self-employment and hypertension among Black women than educational attainment.

The results of this study have important implications. Structural racism limits self-employment among Black Americans through racial segregation (73), restricted family wealth (36, 37, 55), and institutional discrimination (74). This is important considering that full-time self-employment is associated with lower odds of hypertension among Black men. However, nuances among those who are employees with some self-employment income should be examined. Many pursue part-time self-employment to enhance income (45). This could have implications for health, particularly among Black men with lower educational attainment. Policies and programs to support this group should be implemented given that it is likely that part-time self-employment is a viable source of additional income. Programs to increase the number of Black entrepreneurs should address these health implications.

There are several strengths to this study. The study used nationally representative data and analyzed measured hypertension. The study also assessed part-time self-employment which has been overlooked in previous studies. However, there are some limitations. The study design was cross-sectional, and causality could not be assessed. Hypertension was defined as having ≥ 140 systolic blood pressure, ≥ 90 diastolic blood pressure, or reporting currently taking anti-hypertensive medication. Contemporary recommendations for assessing hypertension have lowered the blood pressure threshold to 130/80 mm Hg (75). However, sensitivity analyses show that the associations between employment categories, educational attainment, and hypertension differ when using the lower blood pressure cutpoints which is likely because more than half of Black NHANES participants would fall into the hypertensive category if defined as blood pressure ≥ 130/80 mm Hg. In NHANES, gender is assessed by the interviewer during the home interview and the only options to identify gender are male or female. Therefore, the dataset is limited in terms of gender identity and including more categories and allowing participants to self-identify their gender may have resulted in different associations. There were no measures in NHANES data to examine the pathways between self-employment and hypertension. Additionally, there were no data on specific characteristics of self-employment. As previously discussed, self-employment characteristics can vary by educational attainment. Those characteristics were not assessed in the data set. The size of the analytical sample may be considered a limitation. Lastly, there was no assessment of racial discrimination in the data set. This is a limitation as racial discrimination is impacts hypertension rates in Black Americans.

In conclusion, the study found full-time self-employment was associated with lower risk of hypertension among Black adults. Employment category was not associated with hypertension among women, but Black men with full-time self-employment had lower risk of hypertension compared to those who were employees with no self-employment income. Educational attainment moderated the association between employment categories and hypertension among Black men A relatively high percentage of Black men who had not graduated from high school and were employees who reported self-employment income had hypertension. These results suggest particularly high rates of hypertension among Black men based on self-employment characteristics and educational attainment. It is critical to identify factors that can exacerbate high rates of hypertension among Black Americans to target effective interventions for hypertension prevention and control. Future studies should examine pathways between part-time self-employment and hypertension rates among Black men with lower educational attainment and seek to understand the gender-related factors that underlie the associations observed among Black men, but not among Black women.

## Data availability statement

Publicly available datasets were analyzed in this study. This data can be found at: https://www.cdc.gov/nchs/nhanes/index.htm.

## Ethics statement

Ethical review and approval was not required for the study on human participants in accordance with the local legislation and institutional requirements. Written informed consent from the participants was obtained by the National Center for Health Statistics.

## Author contributions

CB was responsible for the conceptualization, data collection, data analyses, and manuscript writing. CT, JO-Y, and RT were responsible for manuscript writing and editing. All authors contributed to the article and approved the submitted version.

## Funding

RT is funded by the National Institute on Minority Health and Health Disparities (U54MD000214) and the National Institute on Aging (K02AG059140).

## Conflict of interest

The authors declare that the research was conducted in the absence of any commercial or financial relationships that could be construed as a potential conflict of interest.

## Publisher's note

All claims expressed in this article are solely those of the authors and do not necessarily represent those of their affiliated organizations, or those of the publisher, the editors and the reviewers. Any product that may be evaluated in this article, or claim that may be made by its manufacturer, is not guaranteed or endorsed by the publisher.
